# Global Association of COVID-19 Pandemic Measures with Cancer Treatment: A Systematic Review and Meta-Analysis

**DOI:** 10.3390/cancers14225490

**Published:** 2022-11-08

**Authors:** Federica Teglia, Marco Angelini, Giulia Casolari, Laura Astolfi, Paolo Boffetta

**Affiliations:** 1 Department of Medical and Surgical Sciences, University of Bologna, 40138 Bologna, Italy; 2 Stony Brook Cancer Center, Stony Brook University, New York, NY 40138, USA

**Keywords:** COVID-19, pandemic, cancer treatment, radiation oncology, cancer drug therapy, oncological surgery, healthcare rationing

## Abstract

**Simple Summary:**

Oncological departments have been profoundly affected by the COVID-19 pandemic. To determine if there has been a decrease in cancer treatment in the COVID-19 era, we analyzed data from 47 studies that reported on the numbers or variations in cancer treatment between the pandemic and pre-pandemic periods. We found a significant reduction in the number of oncological surgery, radiotherapy, and systemic therapies for cancer patients. These findings suggest that increased cancer-related mortality may occur, requiring public health strategies to limit this.

**Abstract:**

Importance: The COVID-19 pandemic has put a serious strain on health services, including cancer treatment. Objective: This study aimed to investigate the changes in cancer treatment worldwide during the first phase of the SARS-CoV-2 outbreak. Data Sources: Pubmed, Proquest, and Scopus databases were searched comprehensively for articles published between 1 January 2020 and 12 December 2021, in order to perform a systematic review and meta-analysis conducted following the PRISMA statement. Study Selection: Studies and articles that reported data on the number of or variation in cancer treatments between the pandemic and pre-pandemic periods, comprising oncological surgery, radiotherapy, and systemic therapies, were included. Data Extraction and Synthesis: Data were extracted from two pairs of independent reviewers. The weighted average of the percentage variation was calculated between the two periods to assess the change in the number of cancer treatments performed during the pandemic. Stratified analyses were performed by type of treatment, geographic area, time period, study setting, and type of cancer. Results: Among the 47 articles retained, we found an overall reduction of −18.7% (95% CI, −24.1 to −13.3) in the total number of cancer treatments administered during the COVID-19 pandemic compared to the previous periods. Surgical treatment had a larger decrease compared to medical treatment (−33.9% versus −12.6%). For all three types of treatments, we identified a U-shaped temporal trend during the entire period January–October 2020. Significant decreases were also identified for different types of cancer, in particular for skin cancer (−34.7% [95% CI, −46.8 to −22.5]) and for all geographic areas, in particular, Asia (−42.1% [95% CI, −49.6 to −34.7]). Conclusions and Relevance: The interruption, delay, and modifications to cancer treatment due to the COVID-19 pandemic are expected to alter the quality of care and patient outcomes.

## 1. Introduction

The COVID-19 pandemic has significantly impacted the management of cancer patients due to the tremendous pressures on the medical system. Oncology departments drastically needed to modify their care systems and established new priorities. To limit viral transmission, different levels of preventive measures were set up [1].

The burden of the pandemic added significant challenges to the complexity of oncological care. Since patients with cancer were at risk of more severe complications from COVID-19 than healthy individuals [2], elective cancer treatments were postponed in most countries in an attempt to balance the risk of contracting COVID-19 and the benefits of cancer treatment in the oncological population [3].

Although the impact of the COVID-19 pandemic on cancer treatment has been documented [4,5], its magnitude and duration have not been quantified in a detailed matter. We performed a systematic review and meta-analysis to analyze the variation in the total number of cancer treatments administered since the beginning of the pandemic compared to the previous period. The objective of this research was to evaluate the magnitude of the effect of the COVID-19 pandemic on cancer treatment and its persistence in time.

## 2. Materials and Methods

### 2.1. Search Strategy and Selection Criteria 

The research protocol used was included in the PROSPERO Register (registration number CRD42022314314) and consisted of a systematic review and meta-analysis conducted according to the PRISMA statement [6]. As this article is part of a larger project that aims to assess the global impact of the COVID-19 pandemic on cancer patients, including not only oncologic treatment but also cancer screening, diagnosis, and medical visits for oncologic patients, the search strategy and selection criteria are the same as those already extensively discussed in the previous paper on cancer screening [7] and to which reference can be made for details. Briefly, we followed the PICO process; conducted a search of PubMed, Proquest, and Scopus; and selected articles reporting quantitative variations in the number of cancer treatments performed before and after the beginning of the COVID-19 pandemic.

### 2.2. Data Collection and Quality Assessment 

The processes of the identification, screening, and inclusion of the articles in our systematic review and meta-analysis have been detailed above [7]. 

For the analysis of cancer treatments, we retained 47 articles: 34 on surgical treatments, 21 on medical treatments (including 10 articles reporting results for both), 1 on hematopoietic cell transplantation, and 1 on unspecified cancer treatments. We classified the types of treatments into two major groups: surgical treatments, including minor and major surgery, and medical treatments, including radiotherapy, chemotherapy, and immunotherapy. We finally performed a quality assessment of all the studies included in our review using the Critical Appraisal Skills Programme (CASP) score for qualitative research [8]; Table S1 lists the studies included in the present analysis, their major characteristics, and the quality assessment.

### 2.3. Statistical Analyses

Statistical analysis is also discussed in detail in the previous article [7]. Simply put, we calculated the weighted average for the number of daily events in the pre-pandemic period. We performed additional analyses by geographical area, type of setting, and period, identifying five time intervals (January–February 2020; March 2020; April 2020; May 2020; June–October 2020). 

Many studies reported data about different cancer sites or periods. In order to avoid counting the same article multiple times, we used the mean value if the variables were reiterated within the same article. For example, if an article reported data for three different cancers, we used their weighted mean to assess the variation when all cancers were considered together. 

We finally fitted multivariate linear models. We considered the funnel plot and performed the Egger’s regression asymmetry test to assess publication bias [9]. No ethics committee approval was necessary because the study was restricted to publicly available data. For all statistical analyses, we used STATA version 16.1 (Stata Corp., College Station, TX, USA). This research was supported by the internal resources of the participating institutions.

## 3. Results

Weighted average variations for overall, surgical, and medical treatments are reported in Table 1, stratified by period, study setting, geographic area, type of cancer, and type of medical treatment.

### 3.1. Treatments Overall

**Figure 1:**
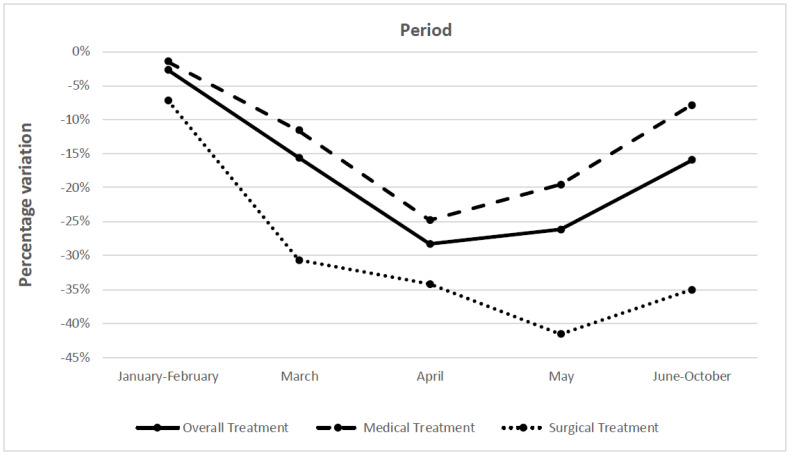
Weighted average variation of overall, medical, and surgical cancer treatment performed from January to October 2020 compared to the pre-pandemic period by time period.

The average variation in oncological treatments throughout January–October 2020 was −18.7% (95% CI, −24.1 to −13.3) compared to the pre-COVID-19 period (Table 1). Analyzing the five periods individually, all of them excluding January-February showed a statistically significant decrease compared to the pre-pandemic period. In particular, the most marked decline was observed in April (−28.3%, 95% CI −37.2 to −19.4), followed by May (−26.2%, 95% CI −34.7 to −17.6) and June-October (−16.0%, 95% CI −27.9 to −4.1) (Table 1 and Figure 1. When stratified by study setting, the weighted average of the treatment variation was −21.5% (95% CI: −31.3 to −11.7) for clinic-based settings and −17.2% (95% CI −23.2 to −11.1) for population-based ones. The mean decrease in cancer treatments was different according to the tumor site and cancer type. The lowest variation was observed for genitourinary cancers (−2.7%, 95% CI −20.6 to 15.1) and lung cancer (−5.2%, 95% CI −15.6 to 5.1) and the highest for skin cancers (−34.7%, 95% CI −46.8 to −22.5). Furthermore, treatment for breast cancer decreased by −18.0% (95% CI, −29.4 to −6.5), cervical cancer by −24.6% (95% CI, −37.5 to −11.6), and prostate cancer by −11.5% (95% CI, −39.0 to 16.0) (Table 1).

We also identified four main geographic areas based on the distribution of the countries analyzed. Figure S1 shows the distribution of the countries in the corresponding geographic areas of the studies included in the analysis and Table 2 shows the results by geographic area. The weighted average variation throughout the entire COVID-19 period was −34.6% for North America, −7.9%, for Europe, −20.3% for Latin America, and −42.1% for Asia. We also performed an analysis by period of each geographic area and the maximum decrease was present in April for North America, Latin America, and Europe, and in May for Asia. The cancer site and the study setting were finally evaluated in each geographic area (Table 2).

### 3.2. Surgical Treatments

Oncologic surgical treatments presented a global reduction of −33.9% (95% CI, −39.9 to −27.9) over the entire time period (Table 1). There was a monotonic decrease up to May, when the minimum value was reached (−41.6% [95% CI, −51.4 to −31.8]) and a significant reduction was still present in June-October (−35.1% [95% CI, −51.6 to −18.6]) (Figure 1). When stratifying for geographic area, Asia showed the largest variation (−45.8% [95% CI, −52.1 to −39.6]) and Europe the smallest (−20.9% [95% CI, −30.1 to −11.7]). Studies with a clinic-based setting reported a larger decrease compared to the population-based ones (−38.1% versus −31.5%). All the cancer groups (genitourinary, gastrointestinal, breast, and skin) showed statistically significant decreases corresponding to −21.6% (95% CI, 31.1 to −12.0), −20.9% (95% CI, −33.9 to −8.0), −26.8% (95% CI, −51.3 to −2.2) and −29.9% (95% CI, −45.3 to −14.4), respectively (Table 1). 

### 3.3. Medical Treatments

The overall percentage variation in the number of medical cancer treatments performed throughout the period January–October 2020 compared to the pre-COVID-19 period was equal to −12.6% (95% CI, −44.9 to −29.7). The temporal trend displayed the maximum decrease in April 2020 (−24.8%, 95% CI: −40.7; −9.0) and a significant reduction was still present in May (−19.6%, 95% CI −30.6 to −8.5) (Figure 1 and Table 1). The two types of oncological medical treatments presented different variations: −6.6% (95% CI, −22.2 to 8.9) for radiotherapy and −18.5% (95% CI, −28.7 to −8.2) for chemotherapy and immunotherapy. The weighted average variation for studies with clinic-based settings experienced a more pronounced decrease (−17.2%, 95% CI −33.0 to −1.5) than the population-based ones (−9.9%, 95% CI −17.5 to −2.3). During the COVID−19 period, in North America, the number of medical treatments decreased by −17.1% (95% CI: −54.4 to 20.2), in Latin America by −18.2% (95% CI: −30.4 to −6.1), in Asia by −36.7% (95% CI, −59.4 to −13.9), and in Europe by −3.8% (95% CI: −15.4 to 7.8). In the stratification of cancer sites, skin cancer presented the largest decrease (−53.5%, 95% CI −83.8 to −23.6) (Table 1). 

Table 3 illustrates the linear regression model for overall, medical, and surgical treatments. A statistical significance is present for surgical treatments that decreased by −27.1% (95% CI, −43.1 to −11.1) using medical treatments as a reference. No significant difference was found between periods, geographic areas, and settings, using January–February, Latin America, and clinic-based settings as references in this analysis.

## 4. Discussion

The present meta-analysis found a significant decrease in the number of overall oncologic treatments performed during the period January–October 2020 compared to the pre-pandemic period. The difficulty in reaching hospitals for appropriate care [10], inability of hospitals to deliver services because of the reallocation of resources to combat the COVID-19 pandemic, consequent reorganization of hospital departments, and immunocompromised nature of cancer patients probably had implications on oncologic patients and services and consequently on the treatment dynamics [4]. In fact, various guidelines for treatment modification were published during that period in order to reduce in-person visits and access to health facilities during the early phase of the COVID-19 pandemic and to mitigate the potential health risks to patients and staff as well as resource shortages [11,12]. For example, the Italian Association of Medical Oncology in March 2020 recommended evaluating case by case the possibility of postponing a treatment, considering the biological aspects of the cancer, clinical characteristics of the patient, and potential risks for COVID-19 infection [13].

Overall oncologic treatment showed a smaller decrease compared to cancer screening tests and diagnoses, which we analyzed elsewhere [7], probably because the decision not to postpone oncological services was influenced by the need to treat diagnosed patients with cancer considering their frailty, whereas the postponement of screening and diagnoses for a population with no diagnosed cancer was seen as less impactful than postponing a treatment. 

The analysis by period revealed a temporal pattern, with a negative peak in April 2020 compared to the previous and subsequent months, identifying a U-shaped trend. The trend was similar for medical and surgical treatments, with the maximum decreases in April and May 2020, respectively. This trend mirrors the lockdown measures adopted by governments in various countries: the Stringency Index of the Oxford COVID-19 Government Response Tracker [14] shows that in those months, COVID-19 measures were implemented globally, with fewer restrictions than in the previous and following months. However, the multilinear regression analysis did not show differences between the periods included in the analysis in contrast with the results of the analyses of screening tests [7] and diagnoses. In fact, while screening tests and cancer diagnoses had their negative peaks in April, as shown in the previous studies of the same research project, oncologic treatments tended to have a slower recovery in the following months. This may be due to the need for re-staging or re-visiting of patients who were previously scheduled for treatment or whose disease worsened during the time when oncological services were reduced or interrupted. Furthermore, safety measures for preventing COVID-19 contamination may have increased the time needed to carry out the procedure and/or the hospital admission.

The stratification of the geographic areas showed that the smallest decrease occurred in Europe compared to North America, Latin America, and Asia, for medical, surgical, and overall treatments. Various countries adopted different guidelines and recommendations regarding cancer patient management during the COVID-19 pandemic [12]. The European Society of Medical Oncology established a clinical benefit scale for categorizing patients into three levels of priority (high, medium, and low) to receive active cancer treatment during the COVID-19 pandemic; in particular, for patients receiving active treatment it was recommended to identify specific pathways to guarantee the timing of the treatment (e.g., prioritize adjuvant therapies in patients with high-risk disease), modify regimen schedules to reduce the number of clinic visits (e.g., three or two weekly as opposed to weekly, oral or subcutaneous vs. IV), and favor phone or web-technology types of contact [15]. The American Society of Clinical Oncology recommended that it was essential to limit hospital access and that physicians should postpone follow-up visits for patients who were not in active cancer treatment and provide patient communication via telemedicine/phone calls [16]. 

The analysis by tumor type showed the greatest decrease for skin cancer (−34.7% overall, −53.5% for medical, and −29.9% for surgical treatments). In a study on melanoma and other skin cancer patients, it was suggested that the treatment of patients with T0 to T1 stages could be delayed by up to three months, providing there was no macroscopic residual disease, and the treatment of patients with tumors T2 or higher could also be delayed by up to 3 months if the biopsy margins were negative [17]. The decrease in the number of treatments can be directly related to the reduction in the number of skin cancer diagnoses, which has been estimated at 68.6% in the UK during the first three months of the pandemic [18]. Furthermore, considering that certain cutaneous malignancies can be managed surgically in the office under local anesthesia, an increase in in-office procedures, which has been reported elsewhere [19], could partially explain the decrease in surgical procedures for skin cancers.

Clinic-based settings presented a slightly larger decrease compared to population-based ones for medical, surgical, and overall treatments, which is consistent with what has been reported in previous studies [7].

On the contrary, our meta-analysis highlighted that cancers with high lethality showed smaller drops in treatment numbers. For example, for lung cancer, one of the most deadly cancers [20], treatments decreased by only −5.2%, and it has been suggested that the treatment of lung cancer patients should not be delayed to prevent rapid cancer progression [21]. 

Among the medical treatments, radiotherapy decreased less than systemic treatments (−6.6% vs. −18.5%). Radiotherapy, which is necessary in up to 50% of patients with cancer and represents 40% of total cancer treatments, has become the treatment of choice in most cases during the COVID-19 pandemic [22]. The suspension of concurrent systemic therapies and radiotherapy in favor of radiotherapy alone was also reported [23], and some published recommendations also encouraged the use of radiation therapy, when appropriate, to delay surgery and inpatient hospitalization [24]. 

An important aspect of our study is the comparison of surgical and medical treatments based on the multivariate analysis, which confirmed that surgical treatments decreased more than medical ones. This may be due to the higher risk of infection for patients and healthcare workers during surgery related to aerosol-generating techniques [25], the subsequent necessity to screen and test for COVID-19 for patients undergoing surgery [26], and the higher mortality risk in cancer patients with COVID-19 compared to the general population [27]. Additional possible explanations for this difference are the continuation of medical treatment even with fewer chemotherapy cycles and, where possible, switching from intravenous to oral treatments, which can be delivered to patients’ homes [28]. Other substantial changes in treatment protocols and procedures were proposed including the modification of dosing schedules or the prioritization of curative intent treatments for the management of cancer patients in the COVID-19 era [12,29,30]. In the surgical field, it was recommended to encourage the use of non-surgical therapies for gynecologic cancers, when appropriate, to delay surgery and inpatient hospitalization [24]. In order to reduce the risk of exposure to SARS-CoV-2 by aerosol-generating procedures, published recommendations suggested undertaking surgery via an open abdominal procedure instead of laparoscopic and robotic surgery [31] and realizing a transition from mostly general anesthesia to regional anesthesia with sedation [32,33], allowing for a reduction in the admission duration. As a consequence, the decrease in scheduled elective surgical operations led to an increase in the proportion of emergency admissions [5], which may heighten concerns about cancer outcomes since they are associated with a worse prognosis [34].

Emerging models predicted a 20% excess in mortality in the oncology population as a result of delays in diagnosis and treatment during this pandemic [35]. As it will take a considerable amount of time to resume full capacity following the pandemic and as at the time of its outbreak, nobody could have predicted its duration, it is plausible that treatment delays and modifications were present also after October 2020, although we were not able to identify published studies. 

The present meta-analysis suffers from some limitations due to the methods of analysis, including the attribution of an observation to one of the five periods based only on its beginning date and the need to impute the number of daily events when it was not reported. 

No evidence of publication bias either qualitatively according to the funnel plot asymmetry or quantitatively according to the Egger’s regression (*p*-values = 0.5 for overall treatment, 0.88 for surgical treatment, and 0.99 for medical treatment) test was identified. 

## 5. Conclusions

In conclusion, the COVID-19 pandemic was one of the greatest public health crises to affect the management and services for oncological patients, including the inability to receive appropriate medical and surgical treatments. In the current meta-analysis, we quantified the decrease in cancer treatments, its temporal trend, and its characteristics by geographical area and cancer type. Furthermore, our study showed a smaller decline in medical treatments compared to surgical treatments. Due to therapeutic delays and the possible worsening of the tumor stage, an increase in cancer mortality is likely to occur and studies will be needed to evaluate this trend. The COVID-19 pandemic has created a large cohort of patients treated with therapeutic schemes different from those in the pre-pandemic period; it is therefore appropriate to correlate these immediate effects with the long-term oncologic outcomes of these patients. Future national, local, and institutional guidelines on cancer care will need to consider the decrease in treatments during the COVID-19 pandemic in order to lessen the likely impact this will have on short- and long-term mortality.

## Figures and Tables

**Table 1:** Weighted percentage differences between COVID-19 and pre-pandemic periods for overall, surgical, and medical cancer treatments by period, geographic area, study setting, type of cancer, and type of medical treatment.

	Percent Difference, % (95% CI)		
Characteristic	Overall Treatment	Surgical Treatment	Medical Treatment
**Total**	−18.7 (−24.1 to −13.3)	−33.9 (−39.9 to −27.9)	−12.6 (−20.4 to −4.8)
**Period (2020)**			
January–February	−2.7 (−10.0 to 4.5)	−7.2 (−23.8 to 9.3)	−1.5 (−8.4 to 5.5)
March	−15.6 (−23.7 to −7.6)	−30.7 (−39.6 to −21.9)	−11.6 (−23.3 to 1.2)
April	−28.3 (−37.2 to −19.4)	−34.2 (−44.5 to −23.9)	−24.8 (−40.7 to −9.0)
May	−26.2 (−34.7 to −17.6)	−41.6 (−51.4 to −31.8)	−19.6 (−30.6 to −8.5)
June–October	−16.0 (27−9 to −4.1)	−35.1 (−51.6 to −18.6)	−7.9 (−23.6 to 7.8)
**Geographic area**			
North America	−34.6 (−47.4 to −21.8)	−39.2 (−53.7 to −24.8)	−17.1 (−54.4 to 20.2)
Europe	−7.9 (−15.6 to −0.2)	−20.9 (−30.1 to −11.7)	−3.8 (−15.4 to 7.8)
Latin America	−20.3 (−31.2 to −9.4)	−38.3 (−54.8 to −21.7)	−18.2 (−30.4 to −6.1)
Asia	−42.1 (−49.6 to −34.7)	−45.8 (−52.1 to −39.6)	−36.7 (−59.4 to −13.9)
**Study setting**			
Clinic-based	−21.5 (−31.3 to −11.7)	−38.1 (−47.5 to −28.7)	−17.2 (−33.0 to −1.5)
Population-based	−17.2 (−23.2 to −11.1)	−31.5 (−40.7 to −22.3)	−9.9 (−17.5 to −2.3)
**Type of cancer**			
Breast	−18.0 (−29.4 to −6.5)	−26.8 (−51.3 to −2.2)	−4.6 (−17.6 to 8.3)
Genitourinary	−2.7 (−20.6 to 15.1)	−20.9 (−33.9 to −8.0)	13.3 (−30.3 to 56.9)
Gastrointestinal	−14.4 (−24.6 to −4.2)	−21.6 (−31.1 to −12.0)	−2.6 (−31.9 to 26.6)
Lung	−5.2 (−15.6 to 5.1)	⸺	⸺
Colorectal	−23.0 (−34.5 to −11.5)	⸺	⸺
Prostate	−11.5 (−39.0 to 16.0)	⸺	⸺
Cervix	−24.6 (−37.5 to −11.6)	⸺	⸺
Skin cancer	−34.7 (−46.8 to −22.5)	−29.9 (−45.3 to −14.4)	−53.5 (−83.3 to −23.6)
**Type of medical treatment**			
Systemic therapy	⸺	⸺	−18.5 (−28.7 to −8.2)
Radiotherapy	⸺	⸺	−6.6 (−22.2 to 8.9)

**Table 2:** Weighted average variation of overall oncological treatment performed from January to October 2020 compared to the pre-pandemic period for North America, Europe, Asia, and Latin America by period, cancer site, and study setting.

	Percent Difference, % (95% CI)			
Cancer Treatments	North America (%, 95% CI)	Europe (%, 95% CI)	Asia (%, 95% CI)	Latin America (%, 95% CI)
**Period**				
January–February	⸺	−0.2 (−8.2 to +7.9)	⸺	⸺
March	−21.6 (−40.3 to −2.9)	−3.6 (−20.2 to 13.0)	−38.7 (−47.2 to −30.2)	−17.1 (−29.2 to −5.0)
April	−39.0 (−68.7 to −9.3)	−20.1 (−30.9 to −9.3)	−53.6 (−70.7 to −36.4)	−43.8 (−94.2 to +6.5)
May	−32.0 (−65.6 to 1.7)	−14.4 (−26.5 to −2.4)	−59.0 (−95.5 to −22.5)	⸺
June–October	−31.5 (−51.5 to −11.6)	−0.6 (−21.1 to 20.0)	⸺	⸺
**Cancer site**				
Gastrointestinal	−31.0 (−91.8 to 29.7)	−8.6 (−19.6 to 2.3)	−47.3 (−71.4 to −23.2)	⸺
Genitourinary	−27.7 (−45.5 to −9.8)	4.9 (−18.6 to 28.5)	⸺	⸺
Skin	−34.9 (−52.0 to −17.7)	−39.9 (−75.9 to −3.9)	⸺	⸺
Breast	−35.0 (−94.6 to 24.6)	−13.2 (−21.0 to −5.5)	⸺	−12.9 (−24.0 to −1.8)
**Study setting**				
Clinic-based	−24.2 (−66.0 to +17.5)	−9.8 (−25.5 to 5.8)	−46.3 (−56.0 to −36.5)	−25.7 (−45.6 to −5.7)
Population-based	−37.2% (−51.5 to −22.9)	−7.0 (−14.8 to 0.9)	−39.2 (−59.8 to −18.6)	−16.0 (−25.2 to −6.8)

**Table 3:** Adjusted differences based on multivariate linear analysis for overall, surgical, and medical cancer treatments by type of treatment, period, geographic area, and study setting.

	Coefficient (95% CI)		
Characteristic	Overall Treatment	Surgical Treatment	Medical Treatment
**Type of treatment**			
Medical treatment	0 [Reference]	⸺	⸺
Surgical treatment	−27.1% (−43.1 to −11.1)	⸺	⸺
**Period (2020)**			
January–February	0 [Reference]	0 [Reference]	0 [Reference]
March	1.0% (−27.7 to 28.7)	−3.1% (−42.5 to 36.3)	3.3% (−41.0 to 47.5)
April	−22.1% (−53.2 to 8.9)	−14.4% (−60.2 to 31.4)	−32.7% (−80.6 to 15.3)
May	−13.7% (−54.2 to 26.9)	−29.1% (−100.0 to 48.7)	−11.6% (−69.0 to 45.8)
June–October	−1.9 (−38.7 to 34.9)	−20.0% (−77.7 to 37.7)	4.3% (−51.3 to 59.8)
**Geographic area**			
North America	0 [Reference]	0 [Reference]	0 [Reference]
Europe	6.6 (−17.6 to 30.9)	2.1% (−26.9 to 31.2)	29.7% (−41.5 to 100.9)
Latin America	−17.5 (−46.4 to 11.4)	−22.1% (−57.8 to 13.7)	3.5% (−70.1 to 77.1)
Asia	−19.5 (−48.0 to 9.0)	−20.0% (−53.0 to 13.0)	−4.8% (−83.0 to 73.4)
**Study setting**			
Clinic-based	0 [Reference]	0 [Reference]	0 [Reference]
Population-based	−2.3(−19.9 to 15.3)	0.9% (−23.3 to 25.1)	−6.4% (−35.9 to 23.1)

## Data Availability

Data used in this study are derived form the studies included in Supplementary Table S1.

## Supplementary Materials

The following supporting information can be downloaded at: https://www.mdpi.com/article/10.3390/cancers14225490/s1, Table S1: Characteristics of selected studies on cancer treatment; Figure S1: Representation of geographic areas of the studies included in the analysis of cancer treatment variations.
