# Supplementary material for: Global Association of COVID-19 Pandemic Measures with Cancer Treatment: A Systematic Review and Meta-Analysis
Source: Cancers (Basel). 2022 Nov 8;14(22):5490. doi: 10.3390/cancers14225490 (PMC9688091; doi:10.3390/cancers14225490)
Supplement: Supplementary file 1 [file cancers-14-05490-s001.zip › cancers-1956126-supplementary.pdf]

Supplementary Materials

Supplementary Table S1 Characteristics of studies selected for cancer treatment

Ref.	Country	Contrast period	Period on exam	Site of cancer	Setting and database	Quality assessment score
(1)	Turkey	March 10, 2019 – May 10, 2019	March 10, 2020 – May 10, 2020	Miscellaneous	Hospital Registration System of Istanbul University, Istitute of Oncology	10
(2)	Turkey	March 15, 2019 – June 1, 2019	March 15, 2020 – June 1, 2020	Miscellaneous	Ankara City Hospital, Ankara Diskapi Yildirim Beyazit Training and Research Hospital, and Gulhane Training and Research Hospital	10
	Turkey	March 15, 2019 – June 1, 2019	March 15, 2020 – June 1, 2020	Miscellaneous	Ankara City Hospital, Ankara Diskapi Yildirim Beyazit Training and Research Hospital, and Gulhane Training and Research Hospital	
(3)	India	January 1, 2020 – January 31, 2020	March 1, 2020 – March 31, 2020	Colorectal	Hospital register	9.5
	India	January 1, 2020 – January 31, 2020	April 1, 2020 – April 30, 2020	Colorectal	Hospital register	9.5
	India	January 1, 2020 – January 31, 2020	May 1, 2020 – May 31, 2020	Colorectal	Hospital register	9.5
	India	January 1, 2020 – January 31, 2020	March 1, 2020 – March 31, 2020	Colorectal	Hospital register	9.5
	India	January 1, 2020 – January 31, 2020	April 1, 2020 – April 30, 2020	Colorectal	Hospital register	9.5
	India	January 1, 2020 – January 31, 2020	May 1, 2020 – May 31, 2020	Colorectal	Hospital register	9.5
	India	January 1, 2020 – January 31, 2020	March 1, 2020 – March 31, 2020	Colorectal	Hospital register	9.5
	India	January 1, 2020 – January 31, 2020	April 1, 2020 – April 30, 2020	Colorectal	Hospital register	9.5
	India	January 1, 2020 – January 31, 2020	May 1, 2020 – May 31, 2020	Colorectal	Hospital register	9.5
(4)	USA	January 1, 2020 – February 29, 2020	March 1, 2020 – April 30, 2020	Miscellaneous*	Rutgers Cancer Institute of New Jersey	10
	USA	January 1, 2020 – February 29, 2020	March 1, 2020 – April 30, 2020	Gynecologic*	Rutgers Cancer Institute of New Jersey	10
	USA	January 1, 2020 – February 29, 2020	March 1, 2020 – April 30, 2020	Gastrointestinal*	Rutgers Cancer Institute of New Jersey	10
	USA	January 1, 2020 – February 29, 2020	March 1, 2020 – April 30, 2020	Melanoma*	Rutgers Cancer Institute of New Jersey	10
	USA	January 1, 2020 – February 29, 2020	March 1, 2020 – April 30, 2020	Breast*	Rutgers Cancer Institute of New Jersey	10
(5)	Canada	March 13, 2019 – August 10, 2019	March 13, 2020 – August 10, 2020	Gastrointestinal	Electronic Health record (EHR) database	10
	Canada	March 13, 2019 – August 10, 2019	March 13, 2020 – August 10, 2020	Gynecologic	Electronic Health record (EHR) database	10

	Canada	March 13, 2019 – August 10, 2019	March 13, 2020 – August 10, 2020	Hematologic cancer	Electronic Health record (EHR) database	10
	Canada	March 13, 2019 – August 10, 2019	March 13, 2020 – August 10, 2020	Head and neck	Electronic Health record (EHR) database	10
	Canada	March 13, 2019 – August 10, 2019	March 13, 2020 – August 10, 2020	Skin cancer	Electronic Health record (EHR) database	10
	Canada	March 13, 2019 – August 10, 2019	March 13, 2020 – August 10, 2020	Miscellaneous	Electronic Health record (EHR) database	10
	Canada	March 13, 2019 – August 10, 2019	March 13, 2020 – August 10, 2020	Lung	Electronic Health record (EHR) database	10
	Canada	March 13, 2019 – August 10, 2019	March 13, 2020 – August 10, 2020	Prostate	Electronic Health record (EHR) database	10
	Canada	March 13, 2019 – August 10, 2019	March 13, 2020 – August 10, 2020	CNS	Electronic Health record (EHR) database	10
	Canada	March 13, 2019 – August 10, 2019	March 13, 2020 – August 10, 2020	Breast	Electronic Health record (EHR) database	10
	Canada	March 13, 2019 – August 10, 2019	March 13, 2020 – August 10, 2020	Genito-urinary	Electronic Health record (EHR) database	10
	Canada	March 13, 2019 – August 10, 2019	March 13, 2020 – August 10, 2020	Miscellaneous	Electronic Health record (EHR) database	10
(6)	France	January 1, 2019 – December 31, 2019	March 16, 2020 – May 12, 2020	Colorectal	National database	9
	France	January 1, 2020 – March 15, 2020	March 16, 2020 – May 12, 2020	Colorectal	National database	9
	France	May 13, 2020 – October 1, 2020	March 16, 2020 – May 12, 2020	Colorectal	National database	9
(7)	Italy	March 9, 2019 – April 15, 2019	March 9, 2020 – April 15, 2020	Colorectal	Department of Surgery, University of Torino, Italy	10
(8)	India	April 1, 2019 – September 30, 2019	April 1, 2020 – September 30, 2020	Miscellaneous	Tertiary care surgical oncology department at an academic hospital in India	9.5
	India	April 1, 2019 – September 30, 2019	April 1, 2020 – September 30, 2020	Miscellaneous	Tertiary care surgical oncology department at an academic hospital in India	9.5

	India	April 1, 2019 – September 30, 2019	April 1, 2020 – September 30, 2020	Head and neck	Tertiary care surgical oncology department at an academic hospital in India	9.5
	India	April 1, 2019 – September 30, 2019	April 1, 2020 – September 30, 2020	Gastrointestinal	Tertiary care surgical oncology department at an academic hospital in India	9.5
	India	April 1, 2019 – September 30, 2019	April 1, 2020 – September 30, 2020	Genito-urinary	Tertiary care surgical oncology department at an academic hospital in India	9.5
	India	April 1, 2019 – September 30, 2019	April 1, 2020 – September 30, 2020	Miscellaneous	Tertiary care surgical oncology department at an academic hospital in India	9.5
(9)	Canada	March 16, 2019 – April 30, 2019	March 16, 2020 – April 30, 2020	Breast	Breast surgery database at Providence Breast Centre	9.5
(10)	India	March 1, 2019 – May 31, 2019	March 1, 2020 – May 31, 2020	Miscellaneous	National Cancer Grid of India	10
	India	March 1, 2019 – May 31, 2019	March 1, 2020 – May 31, 2020	Miscellaneous	National Cancer Grid of India	10
	India	March 1, 2019 – May 31, 2019	March 1, 2020 – May 31, 2020	Miscellaneous	National Cancer Grid of India	10
	India	March 1, 2019 – May 31, 2019	March 1, 2020 – May 31, 2020	Miscellaneous	National Cancer Grid of India	10
(11)	USA	March 1, 2019 – March 31, 2019	March 1, 2020 – March 31, 2020	Miscellaneous*	Proprietary provider clearinghouse registry	9.5
	USA	March 1, 2019 – March 31, 2019	March 1, 2020 – March 31, 2020	Breast*	Proprietary provider clearinghouse registry	9.5
	USA	March 1, 2019 – March 31, 2019	March 1, 2020 – March 31, 2020	Colorectal*	Proprietary provider clearinghouse registry	9.5
	USA	March 1, 2019 – March 31, 2019	March 1, 2020 – March 31, 2020	Prostate*	Proprietary provider clearinghouse registry	9.5
	USA	July 1, 2019 – July 31, 2019	July 1, 2020 – July 31, 2020	Miscellaneous*	Proprietary provider clearinghouse registry	9.5
	USA	July 1, 2019 – July 31, 2019	July 1, 2020 – July 31, 2020	Breast*	Proprietary provider clearinghouse registry	9.5
	USA	July 1, 2019 – July 31, 2019	July 1, 2020 – July 31, 2020	Colorectal*	Proprietary provider clearinghouse registry	9.5
	USA	July 1, 2019 – July 31, 2019	July 1, 2020 – July 31, 2020	Prostate*	Proprietary provider clearinghouse registry	9.5
	USA	April 1, 2019 – April 30, 2019	April 1, 2020 – April 30, 2020	Miscellaneous*	Proprietary provider clearinghouse registry	9.5

	USA	April 1, 2019 – April 30, 2019	April 1, 2020 – April 30, 2020	Breast*	Proprietary provider clearinghouse registry	9.5
	USA	April 1, 2019 – April 30, 2019	April 1, 2020 – April 30, 2020	Colorectal*	Proprietary provider clearinghouse registry	9.5
	USA	April 1, 2019 – April 30, 2019	April 1, 2020 – April 30, 2020	Prostate*	Proprietary provider clearinghouse registry	9.5
	USA	May 1, 2019 – May 31, 2019	May 1, 2020 – May 31, 2020	Miscellaneous*	Proprietary provider clearinghouse registry	9.5
	USA	May 1, 2019 – May 31, 2019	May 1, 2020 – May 31, 2020	Breast*	Proprietary provider clearinghouse registry	9.5
	USA	May 1, 2019 – May 31, 2019	May 1, 2020 – May 31, 2020	Colorectal*	Proprietary provider clearinghouse registry	9.5
	USA	May 1, 2019 – May 31, 2019	May 1, 2020 – May 31, 2020	Prostate*	Proprietary provider clearinghouse registry	9.5
	USA	June 1, 2019 – June 30, 2019	June1, 2020 – June 30, 2020	Miscellaneous*	Proprietary provider clearinghouse registry	9.5
	USA	June 1, 2019 – June 30, 2019	June1, 2020 – June 30, 2020	Breast*	Proprietary provider clearinghouse registry	9.5
	USA	June 1, 2019 – June 30, 2019	June1, 2020 – June 30, 2020	Colorectal*	Proprietary provider clearinghouse registry	9.5
	USA	June 1, 2019 – June 30, 2019	June1, 2020 – June 30, 2020	Prostate*	Proprietary provider clearinghouse registry	9.5
(12)	Brazil	March 1, 2019 – June 30, 2019	March 1, 2020 – June 30, 2020	Head and neck	Public archives of the Hospital Information System of the Brazil's Unified Health System (SIH/SUS) and Outpatient Information System (SUS-SAI/SUS) from Department of Informatics of the SUS	7.5
	Brazil	March 1, 2019 – June 30, 2019	March 1, 2020 – June 30, 2020	Head and neck	Public archives of the Hospital Information System of the Brazil's Unified Health System (SIH/SUS) and Outpatient Information System (SUS-SAI/SUS) from Department of Informatics of the SUS	7.5
(13)	Uruguay	March 1, 2019 – June 30, 2019	March 1, 2020 – June 30, 2020	Breast	Mastology Unit of the School of Medicine, Unidad Docente Assistencial de Mastologia (UDAM)	7.5
(14)	Italy	March 9, 2019 – August 31, 2019	March 9, 2020 – August 31, 2020	Thyroid	Aggregated data from 28 Italian surgical units	7.5
(15)	UK	January 1, 2019 – December 31, 2019	January 1, 2020 – January 31, 2020	Colorectal	National Health Service (NHS) population-based datasets	7
	UK	January 1, 2019 – December 31, 2019	February 1, 2020 - February 29, 2020	Colorectal	National Health Service (NHS) population-based datasets	7
	UK	January 1, 2019 – December 31, 2019	March 1, 2020 – March 31, 2020	Colorectal	National Health Service (NHS) population-based datasets	7

	UK	January 1, 2019 – December 31, 2019	April 1, 2020 – April 30, 2020	Colorectal	National Health Service (NHS) population-based datasets	7
	UK	January 1, 2019 – December 31, 2019	May 1, 2020 – May 31, 2020	Colorectal	National Health Service (NHS) population-based datasets	7
	UK	January 1, 2019 – December 31, 2019	June 1, 2020 – June 30, 2020	Colorectal	National Health Service (NHS) population-based datasets	7
	UK	January 1, 2019 – December 31, 2019	July 1, 2020 – July 31, 2020	Colorectal	National Health Service (NHS) population-based datasets	7
	UK	January 1, 2019 – December 31, 2019	August 1, 2020 – August 31, 2020	Colorectal	National Health Service (NHS) population-based datasets	7
	UK	January 1, 2019 – December 31, 2019	September 1, 2020 – September 30, 2020	Colorectal	National Health Service (NHS) population-based datasets	7
	UK	January 1, 2019 – December 31, 2019	October 1, 2020 – October 31, 2020	Colorectal	National Health Service (NHS) population-based datasets	7
	UK	January 1, 2019 – December 31, 2019	January 1, 2020 – January 31, 2020	Colorectal	National Health Service (NHS) population-based datasets	7
	UK	January 1, 2019 – December 31, 2019	February 1, 2020 - February 29, 2020	Colorectal	National Health Service (NHS) population-based datasets	7
	UK	January 1, 2019 – December 31, 2019	March 1, 2020 – March 31, 2020	Colorectal	National Health Service (NHS) population-based datasets	7
	UK	January 1, 2019 – December 31, 2019	April 1, 2020 – April 30, 2020	Colorectal	National Health Service (NHS) population-based datasets	7
	UK	January 1, 2019 – December 31, 2019	May 1, 2020 – May 31, 2020	Colorectal	National Health Service (NHS) population-based datasets	7
	UK	January 1, 2019 – December 31, 2019	June 1, 2020 – June 30, 2020	Colorectal	National Health Service (NHS) population-based datasets	7
	UK	January 1, 2019 – December 31, 2019	July 1, 2020 – July 31, 2020	Colorectal	National Health Service (NHS) population-based datasets	7
	UK	January 1, 2019 – December 31, 2019	August 1, 2020 – August 31, 2020	Colorectal	National Health Service (NHS) population-based datasets	7
	UK	January 1, 2019 – December 31, 2019	September 1, 2020 – September 30, 2020	Colorectal	National Health Service (NHS) population-based datasets	7
	UK	January 1, 2019 – December 31, 2019	October 1, 2020 – October 31, 2020	Colorectal	National Health Service (NHS) population-based datasets	7
(16)	Italy	March 11, 2019 – May 05, 2019	March 09, 2020– May 04, 2020	Colorectal*	Eight tertiary centres, representative of Northern and Southern Italy	9.5

(17)	Germany	April 01, 2019 – June 30, 2019	April 01, 2020 – June 30, 2020	Lung*	BARMER Ersatzkasse (one of Germany’s leading providers of statutory health insurance)	8
	Germany	April 01, 2019 – June 30, 2019	April 01, 2020 – June 30, 2020	Breast*	BARMER Ersatzkasse (one of Germany’s leading providers of statutory health insurance)	8
	Germany	April 01, 2019 – June 30, 2019	April 01, 2020 – June 30, 2020	Esophageal*	BARMER Ersatzkasse (one of Germany’s leading providers of statutory health insurance)	8
	Germany	April 01, 2019 – June 30, 2019	April 01, 2020 – June 30, 2020	Stomach*	BARMER Ersatzkasse (one of Germany’s leading providers of statutory health insurance)	8
	Germany	April 01, 2019 – June 30, 2019	April 01, 2020 – June 30, 2020	Pancreas*	BARMER Ersatzkasse (one of Germany’s leading providers of statutory health insurance)	8
	Germany	April 01, 2019 – June 30, 2019	April 01, 2020 – June 30, 2020	Colon only*	BARMER Ersatzkasse (one of Germany’s leading providers of statutory health insurance)	8
	Germany	April 01, 2019 – June 30, 2019	April 01, 2020 – June 30, 2020	Rectum only*	BARMER Ersatzkasse (one of Germany’s leading providers of statutory health insurance)	8
	Germany	April 01, 2019 – June 30, 2019	April 01, 2020 – June 30, 2020	Kidney*	BARMER Ersatzkasse (one of Germany’s leading providers of statutory health insurance)	8
	Germany	April 01, 2019 – June 30, 2019	April 01, 2020 – June 30, 2020	Prostate*	BARMER Ersatzkasse (one of Germany’s leading providers of statutory health insurance)	8
(18)	Spain	February 25, 2019 – June 15, 2019	February 25, 2020 – June 15, 2020	Hematologic cancer	La Paz University Hospital (Hematology Unit)	7.5
(19)	Israel	March 14, 2019 – April 30, 2019	March 14, 2020 – April 30, 2020	Melanoma	Rambam Health Care Campus (RHCC)	8.5
(20)	Brazil	March 09, 2019 – May 30, 2019	March 09, 2020 – May 30, 2020	Breast*	AC Camargo Cancer Center (Breast Surgery Department)	7.5
(21)	Italy	March 09, 2019 – May 08, 2019	March 09, 2020 – May 08, 2020	Miscellaneous	Pathological reporting software (Winsap, Engineering, Rome, Italy) of the Pathology Units of “Città della Salute e della Scienza di Torino” University Hospital of Turin	9
	Italy	March 09, 2019 – May 08, 2019	March 09, 2020 – May 08, 2020	Lung	Pathological reporting software (Winsap, Engineering, Rome, Italy) of the Pathology Units of “Città della Salute e della Scienza di Torino” University Hospital of Turin	9
	Italy	March 09, 2019 – May 08, 2019	March 09, 2020 – May 08, 2020	Prostate	Pathological reporting software (Winsap, Engineering, Rome, Italy) of the Pathology Units of “Città della Salute e della Scienza di Torino” University Hospital of Turin	9
	Italy	March 09, 2019 – May 08, 2019	March 09, 2020 – May 08, 2020	Colorectal	Pathological reporting software (Winsap, Engineering, Rome, Italy) of the Pathology Units of “Città della Salute e della Scienza di Torino” University Hospital of Turin	9
	Italy	March 09, 2019 – May 08, 2019	March 09, 2020 – May 08, 2020	Breast	Pathological reporting software (Winsap, Engineering, Rome, Italy) of the Pathology Units of “Città della Salute e della Scienza di Torino” University Hospital of Turin	9
(22)	Chile	March 16, 2019 – June 07, 2019	March 16, 2020 – June 07, 2020	Genito-Urinary	Database of urological oncological surgery	7.5
(23)	UK	March 23, 2019 – June 07, 2019	March 23, 2020 – June 07, 2020	Colorectal	Private Trust	8.5

(24)	Italy	February 24, 2019 – May 08, 2019	February 24, 2020 – May 08, 2020	Miscellaneous	Tertiary care center in Northern Italy	7.5
	Italy	February 24, 2019 – May 08, 2019	February 24, 2020 – May 08, 2020	Colorectal	Tertiary care center in Northern Italy	7.5
(25)	France	January 20, 2020 – March 15, 2020	March 16, 2020 – May 10, 2020	Miscellaneous	The Oscar Lambret Cancer center (Comprehensive Cancer Center of Northern France)	9
	France	January 20, 2020 – March 15, 2020	May 11, 2020 – July 06, 2020	Miscellaneous	The Oscar Lambret Cancer center (Comprehensive Cancer Center of Northern France)	9
	France	January 20, 2020 – March 15, 2020	March 16, 2020 – May 10, 2020	Miscellaneous	The Oscar Lambret Cancer center (Comprehensive Cancer Center of Northern France)	9
	France	January 20, 2020 – March 15, 2020	May 11, 2020 – July 06, 2020	Miscellaneous	The Oscar Lambret Cancer center (Comprehensive Cancer Center of Northern France)	9
	France	January 20, 2020 – March 15, 2020	March 16, 2020 – May 10, 2020	Miscellaneous	The Oscar Lambret Cancer center (Comprehensive Cancer Center of Northern France)	9
	France	January 20, 2020 – March 15, 2020	May 11, 2020 – July 06, 2020	Miscellaneous	The Oscar Lambret Cancer center (Comprehensive Cancer Center of Northern France)	9
(26)	India	October 01, 2019 – February 29, 2020	March 01, 2020 – July 31, 2020	Miscellaneous	Royapettah cancer hospital	7.5
	India	October 01, 2019 – February 29, 2020	March 01, 2020 – July 31, 2020	Miscellaneous	Royapettah cancer hospital	7.5
(27)	Italy	February 01, 2019 – April 30, 2019	February 01, 2020 – April 30, 2020	Breast	Fondazione Policlinico Agostino Gemelli-IRCCS, Rome	7
(28)	Italy	February 01, 2019 – April 30, 2019	February 01, 2020 – April 30, 2020	Gynecologic	Fondazione Policlinico Agostino Gemelli-IRCCS, Rome	9.5
(29)	Italy	June 01, 2019 – September 30, 2019	June 01, 2020 – September 30, 2020	Lung	IEO, European Institute of Oncology IRCCS	10
(30)	USA	January 01, 2020 – February 29, 2020	March 01, 2020 – April 30, 2020	Skin cancer	Plastic Surgical Treatment Centre (PSTC)	8
(31)	Sweden	March 18, 2019 – June 02, 2019	March 18, 2020 – June 02, 2020	Prostate	Swedish Cancer Registry	9.5
	Sweden	March 18, 2019 – June 02, 2019	March 18, 2020 – June 02, 2020	Prostate	Swedish Cancer Registry	9.5
(32)	Italy	February 01, 2019 – March 31, 2019	February 01, 2020 – March 31, 2020	Miscellaneous	Radiation Oncology Unit, Radiotherapy Department, Santa Maria della Misericordia Hospital, ULSS5, Rovigo, Italy	9
(33)	Italy	March 09, 2019 – May 31, 2019	March 09, 2020 – May 31, 2020	Miscellaneous	Operative Unit of Radiotherapy (OUR) of the University Hospital of Pisa	9.5
	Italy	March 09, 2019 – May 31, 2019	March 09, 2020 – May 31, 2020	Miscellaneous	Operative Unit of Radiotherapy (OUR) of the University Hospital of Pisa	9.5
	Italy	March 09, 2019 – May 31, 2019	March 09, 2020 – May 31, 2020	Melanoma	Operative Unit of Radiotherapy (OUR) of the University Hospital of Pisa	9.5
	Italy	March 09, 2019 – May 31, 2019	March 09, 2020 – May 31, 2020	Breast	Operative Unit of Radiotherapy (OUR) of the University Hospital of Pisa	9.5
	Italy	March 09, 2019 – May 31, 2019	March 09, 2020 – May 31, 2020	CNS	Operative Unit of Radiotherapy (OUR) of the University Hospital of Pisa	9.5
(34)	Italy	March 09, 2019 – December 23, 2019	March 09, 2020 – December 23, 2020	Endocrine	Padua Endocrine Surgery Unit	8.5
	Italy	March 09, 2019 – December 23, 2019	March 09, 2020 –	Thyroid	Padua Endocrine Surgery Unit	8.5

			December 23, 2020			
(35)	Australia	April 01, 2019 – June 30, 2019	January 01, 2020 – March 31, 2020	Skin cancer*	MedicineInsight and national Medicare Benefits Schedule (MBS)	7.5
(36)	England	January 01, 2019 – June 30, 2019	January 01, 2020 – June 30, 2020	Breast	National Health Service (NHS) cancer service activity	8.5
	England	January 01, 2019 – January 31, 2019	January 01, 2020 – January 31, 2020	Breast	National Health Service (NHS) cancer service activity	8.5
	England	February 01, 2019 – February 28, 2019	February 01, 2020 – February 29, 2020	Breast	National Health Service (NHS) cancer service activity	8.5
	England	March 01, 2019 – March 31, 2019	March 01, 2020 – March 31, 2020	Breast	National Health Service (NHS) cancer service activity	8.5
	England	April 01, 2019 – April 30, 2019	April 01, 2020 – April 30, 2020	Breast	National Health Service (NHS) cancer service activity	8.5
	England	May 01, 2019 – May 31, 2019	May 01, 2020 – May 31, 2020	Breast	National Health Service (NHS) cancer service activity	8.5
	England	June 01, 2019 – June 30, 2019	June 01, 2020 – June 30, 2020	Breast	National Health Service (NHS) cancer service activity	8.5
(37)	England	September 01, 2019 – February 29, 2020	April 01, 2020 – April 30, 2020	Miscellaneous	Central National Health Service England web database	9.5
	England	September 01, 2019 – February 29, 2020	May 01, 2020 – May 31, 2020	Miscellaneous	Central National Health Service England web database	9.5
	England	September 01, 2019 – February 29, 2020	June 01, 2020 – June 30, 2020	Miscellaneous	Central National Health Service England web database	9.5
	England	September 01, 2019 – February 29, 2020	April 01, 2020 – April 30, 2020	Miscellaneous	Central National Health Service England web database	9.5
	England	September 01, 2019 – February 29, 2020	May 01, 2020 – May 31, 2020	Miscellaneous	Central National Health Service England web database	9.5
	England	September 01, 2019 – February 29, 2020	June 01, 2020 – June 30, 2020	Miscellaneous	Central National Health Service England web database	9.5
	England	September 01, 2019 – February 29, 2020	April 01, 2020 – April 30, 2020	Miscellaneous	Central National Health Service England web database	9.5
	England	September 01, 2019 – February 29, 2020	May 01, 2020 – May 31, 2020	Miscellaneous	Central National Health Service England web database	9.5
	England	September 01, 2019 – February 29, 2020	June 01, 2020 – June 30, 2020	Miscellaneous	Central National Health Service England web database	9.5
	England	September 01, 2019 – February 29, 2020	April 01, 2020 – April 30, 2020	Miscellaneous	Central National Health Service England web database	9.5
	England	September 01, 2019 – February 29, 2020	May 01, 2020 – May 31, 2020	Miscellaneous	Central National Health Service England web database	9.5
	England	September 01, 2019 – February 29, 2020	June 01, 2020 – June 30, 2020	Miscellaneous	Central National Health Service England web database	9.5
	England	September 01, 2019 – February 29, 2020	April 01, 2020 – April 30, 2020	Breast	Central National Health Service England web database	9.5
	England	September 01, 2019 – February 29, 2020	April 01, 2020 – April 30, 2020	Prostate	Central National Health Service England web database	9.5
	England	September 01, 2019 – February 29, 2020	April 01, 2020 – April 30, 2020	Lung	Central National Health Service England web database	9.5

	England	September 01, 2019 – February 29, 2020	April 01, 2020 – April 30, 2020	Skin cancer	Central National Health Service England web database	9.5
	England	September 01, 2019 – February 29, 2020	June 01, 2020 – June 30, 2020	Prostate	Central National Health Service England web database	9.5
	England	September 01, 2019 – February 29, 2020	June 01, 2020 – June 30, 2020	Lung	Central National Health Service England web database	9.5
(38)	UK	April 01, 2019 – April 30, 2019	April 01, 2020 – April 30, 2020	Miscellaneous*	National Radiotherapy Dataset	9
	UK	May 01, 2019 – May 31, 2019	May 01, 2020 – May 31, 2020	Miscellaneous*	National Radiotherapy Dataset	9
	UK	June 01, 2019 – June 30, 2019	June 01, 2020 – June 30, 2020	Miscellaneous*	National Radiotherapy Dataset	9
	UK	April 01, 2019 – April 30, 2019	April 01, 2020 – April 30, 2020	Anal*	National Radiotherapy Dataset	9
	UK	May 01, 2019 – May 31, 2019	May 01, 2020 – May 31, 2020	Anal*	National Radiotherapy Dataset	9
	UK	June 01, 2019 – June 30, 2019	June 01, 2020 – June 30, 2020	Anal*	National Radiotherapy Dataset	9
	UK	April 01, 2019 – April 30, 2019	April 01, 2020 – April 30, 2020	Bladder*	National Radiotherapy Dataset	9
	UK	May 01, 2019 – May 31, 2019	May 01, 2020 – May 31, 2020	Bladder*	National Radiotherapy Dataset	9
	UK	June 01, 2019 – June 30, 2019	June 01, 2020 – June 30, 2020	Bladder*	National Radiotherapy Dataset	9
	UK	April 01, 2019 – April 30, 2019	April 01, 2020 – April 30, 2020	CNS*	National Radiotherapy Dataset	9
	UK	May 01, 2019 – May 31, 2019	May 01, 2020 – May 31, 2020	CNS*	National Radiotherapy Dataset	9
	UK	June 01, 2019 – June 30, 2019	June 01, 2020 – June 30, 2020	CNS*	National Radiotherapy Dataset	9
	UK	April 01, 2019 – April 30, 2019	April 01, 2020 – April 30, 2020	Cervix*	National Radiotherapy Dataset	9
	UK	May 01, 2019 – May 31, 2019	May 01, 2020 – May 31, 2020	Cervix*	National Radiotherapy Dataset	9
	UK	June 01, 2019 – June 30, 2019	June 01, 2020 – June 30, 2020	Cervix*	National Radiotherapy Dataset	9
	UK	April 01, 2019 – April 30, 2019	April 01, 2020 – April 30, 2020	Head and Neck*	National Radiotherapy Dataset	9
	UK	May 01, 2019 – May 31, 2019	May 01, 2020 – May 31, 2020	Head and Neck*	National Radiotherapy Dataset	9
	UK	June 01, 2019 – June 30, 2019	June 01, 2020 – June 30, 2020	Head and Neck*	National Radiotherapy Dataset	9
	UK	April 01, 2019 – April 30, 2019	April 01, 2020 – April 30, 2020	Lung*	National Radiotherapy Dataset	9
	UK	May 01, 2019 – May 31, 2019	May 01, 2020 – May 31, 2020	Lung*	National Radiotherapy Dataset	9
	UK	June 01, 2019 – June 30, 2019	June 01, 2020 – June 30, 2020	Lung*	National Radiotherapy Dataset	9
	UK	April 01, 2019 – April 30, 2019	April 01, 2020 – April 30, 2020	Breast*	National Radiotherapy Dataset	9

	UK	May 01, 2019 – May 31, 2019	May 01, 2020 – May 31, 2020	Breast*	National Radiotherapy Dataset	9
	UK	June 01, 2019 – June 30, 2019	June 01, 2020 – June 30, 2020	Breast*	National Radiotherapy Dataset	9
	UK	April 01, 2019 – April 30, 2019	April 01, 2020 – April 30, 2020	Esophageal*	National Radiotherapy Dataset	9
	UK	May 01, 2019 – May 31, 2019	May 01, 2020 – May 31, 2020	Esophageal*	National Radiotherapy Dataset	9
	UK	June 01, 2019 – June 30, 2019	June 01, 2020 – June 30, 2020	Esophageal*	National Radiotherapy Dataset	9
	UK	April 01, 2019 – April 30, 2019	April 01, 2020 – April 30, 2020	Prostate*	National Radiotherapy Dataset	9
	UK	May 01, 2019 – May 31, 2019	May 01, 2020 – May 31, 2020	Prostate*	National Radiotherapy Dataset	9
	UK	June 01, 2019 – June 30, 2019	June 01, 2020 – June 30, 2020	Prostate*	National Radiotherapy Dataset	9
	UK	April 01, 2019 – April 30, 2019	April 01, 2020 – April 30, 2020	Colorectal*	National Radiotherapy Dataset	9
	UK	May 01, 2019 – May 31, 2019	May 01, 2020 – May 31, 2020	Colorectal*	National Radiotherapy Dataset	9
	UK	June 01, 2019 – June 30, 2019	June 01, 2020 – June 30, 2020	Colorectal*	National Radiotherapy Dataset	9
	UK	April 01, 2019 – April 30, 2019	April 01, 2020 – April 30, 2020	Skin cancer*	National Radiotherapy Dataset	9
	UK	May 01, 2019 – May 31, 2019	May 01, 2020 – May 31, 2020	Skin cancer*	National Radiotherapy Dataset	9
	UK	June 01, 2019 – June 30, 2019	June 01, 2020 – June 30, 2020	Skin cancer*	National Radiotherapy Dataset	9
(39)	Croatia	March 01, 2019 – May 31, 2019	March 01, 2020 – May 31, 2020	Skin cancer	Maxillofacial and Oral Surgery Department (Department), Osijek University Hospital	9.5
(40)	Italy	March 09, 2019 – May 05, 2019	March 09, 2020 – May 05, 2020	Cervix	University Hospital of Bologna, Italy (Gynaecologic Oncology Unit)	9.5
	Italy	March 09, 2019 – May 05, 2019	March 09, 2020 – May 05, 2020	Gynecologic	University Hospital of Bologna, Italy (Gynaecologic Oncology Unit)	9.5
	Italy	March 09, 2019 – May 05, 2019	March 09, 2020 – May 05, 2020	Gynecologic	University Hospital of Bologna, Italy (Gynaecologic Oncology Unit)	9.5
	Italy	March 09, 2019 – May 05, 2019	March 09, 2020 – May 05, 2020	Gynecologic	University Hospital of Bologna, Italy (Gynaecologic Oncology Unit)	9.5
	Italy	March 09, 2019 – May 05, 2019	March 09, 2020 – May 05, 2020	Gynecologic	University Hospital of Bologna, Italy (Gynaecologic Oncology Unit)	9.5
(41)	USA	April 28, 2019 – July 31, 2019	April 28, 2020 – July 31, 2020	Skin cancer	Department of Dermatology, Beth Israel Deaconess Medical Center, Boston	7.5
	USA	April 28, 2019 – July 31, 2019	April 28, 2020 – July 31, 2020	Melanoma	Department of Dermatology, Beth Israel Deaconess Medical Center, Boston	7.5

	USA	April 28, 2019 – July 31, 2019	April 28, 2020 – July 31, 2020	Scc	Department of Dermatology, Beth Israel Deaconess Medical Center, Boston	7.5
	USA	April 28, 2019 – July 31, 2019	April 28, 2020 – July 31, 2020	Bcc	Department of Dermatology, Beth Israel Deaconess Medical Center, Boston	7.5
(42)	South Corea	January 28, 2019 – July 31, 2019	January 28, 2020 – July 31, 2020	Breast	Department of Radiation Oncology, Ajou University School of Medicine	9.5
(43)	Italy	January 01, 2020 – March 08, 2020	March 09, 2020 – April 30, 2020	Solid cancer	Department of Oncology, Mater Salutis Hospital, Legnago (VR)	8.5
(44)	Canada	March 1, 2019 – March 31, 2019	March 1, 2020 – March 31, 2020	Cervix*	CytoBase	9.5
	Canada	April 1, 2019 – April 30, 2019	April 1, 2020 – April 30, 2020	Cervix*	CytoBase	9.5
	Canada	May 1, 2019 – May 31, 2019	May 1, 2020 – May 31, 2020	Cervix*	CytoBase	9.5
	Canada	June 1, 2019 – June 30, 2019	June 1, 2020 – June 30, 2020	Cervix*	CytoBase	9.5
	Canada	July 1, 2019 – July 31, 2019	July 1, 2020 – July 31, 2020	Cervix*	CytoBase	9.5
	Canada	August 1, 2019 – August 31, 2019	August 1, 2020 – August 31, 2020	Cervix*	CytoBase	9.5
(45)	Uruguay	March 16, 2019 – April 30, 2019	March 16, 2020 – April 30, 2020	Miscellaneous*	Public Provider	9.5
	Uruguay	May 01, 2019 – May 31, 2019	May 01, 2020 – May 31, 2020	Miscellaneous*	Public Provider	9.5
	Uruguay	June 01, 2019 – June 30, 2019	June 01, 2020 – June 30, 2020	Miscellaneous*	Public Provider	9.5
	Uruguay	March 16, 2019 – June 30, 2019	March 16, 2020 – June 30, 2020	Miscellaneous*	National cancer institute	9.5
	Chile	April 01, 2019 – April 30, 2019	April 01, 2020 – April 30, 2020	Miscellaneous*	National cancer institute	9.5
	Chile	May 01, 2019 – May 31, 2019	May 01, 2020 – May 31, 2020	Miscellaneous*	National cancer institute	9.5
	Chile	June 01, 2019 – June 30, 2019	June 01, 20120 – June 30, 2020	Miscellaneous*	National cancer institute	9.5
	Chile	April 01, 2019 – April 30, 2019	April 01, 2020 – April 30, 2020	Miscellaneous*	National cancer institute	9.5
	Chile	May 01, 2019 – May 31, 2019	May 01, 2020 – May 31, 2020	Miscellaneous*	National cancer institute	9.5
	Chile	June 01, 2019 – June 30, 2019	June 01, 20120 – June 30, 2020	Miscellaneous*	National cancer institute	9.5
	Chile	March 16, 2019 – April 30, 2019	March 16, 20120 – April 30, 2020	Miscellaneous*	National cancer institute	9.5

	Chile	May 1, 2019 – May 31, 2019	May 1, 2020 – May 31, 2020	Miscellaneous*	National cancer institute	9.5
	Chile	June 01, 2019 – June 30, 2019	June 01, 2020 – June 30, 2020	Miscellaneous*	National cancer institute	9.5
	Mexico	April 01, 2019 – April 30, 2019	April 01, 2020 – April 30, 2020	Miscellaneous*	General Hospital and National Cancer institute	9.5
	Mexico	May 01, 2019 – May 31, 2019	May 01, 2020 – May 31, 2020	Miscellaneous*	General Hospital and National Cancer institute	9.5
	Mexico	June 01, 2019 – June 30, 2019	June 01, 20120 – June 30, 2020	Miscellaneous*	General Hospital and National Cancer institute	9.5
	Mexico	April 01, 2019 – April 30, 2019	April 01, 2020 – April 30, 2020	Miscellaneous*	General Hospital and National Cancer institute	9.5
	Mexico	May 01, 2019 – May 31, 2019	May 01, 2020 – May 31, 2020	Miscellaneous*	General Hospital and National Cancer institute	9.5
	Mexico	June 01, 2019 – June 30, 2019	June 01, 20120 – June 30, 2020	Miscellaneous*	General Hospital and National Cancer institute	9.5
	Mexico	March 16, 2019 – June 30, 2019	March 16, 2020 – June 30, 2020	Miscellaneous*	General Hospital	9.5
	Mexico	April 01, 2019 – April 30, 2019	April 01, 2020 – April 30, 2020	Miscellaneous*	General Hospital and National Cancer institute	9.5
	Mexico	May 01, 2019 – May 31, 2019	May 01, 2020 – May 31, 2020	Miscellaneous*	General Hospital and National Cancer institute	9.5
	Mexico	June 01, 2019 – June 30, 2019	June 01, 20120 – June 30, 2020	Miscellaneous*	General Hospital and National Cancer institute	9.5
	Mexico	April 01, 2019 – April 30, 2019	April 01, 2020 – April 30, 2020	Miscellaneous*	General Hospital and National Cancer institute	9.5
	Mexico	May 01, 2019 – May 31, 2019	May 01, 2020 – May 31, 2020	Miscellaneous*	General Hospital and National Cancer institute	9.5
	Mexico	June 01, 2019 – June 30, 2019	June 01, 20120 – June 30, 2020	Miscellaneous*	General Hospital and National Cancer institute	9.5
	Colombia	March 16, 2019 – April 30, 2019	March 16, 2020 – April 30, 2020	Miscellaneous*	Private Provider	9.5
	Colombia	May 01, 2019 – May 31, 2019	May 01, 2020 – May 31, 2020	Miscellaneous*	Private Provider	9.5
	Colombia	June 01, 2019 – June 30, 2019	June 01, 20120 – June 30, 2020	Miscellaneous*	Private Provider	9.5
	Colombia	March 16, 2019 – April 30, 2019	March 16, 2020 – April 30, 2020	Miscellaneous*	Private Provider	9.5

	Colombia	May 01, 2019 – May 31, 2019	May 01, 2020 – May 31, 2020	Miscellaneous*	Private Provider	9.5
	Colombia	May 01, 2019 – May 31, 2019	June 01, 20120 – June 30, 2020	Miscellaneou	Private Provider	9.5
	Perù	March 16, 2019 – April 30, 2019	March 16, 2020 – April 30, 2020	Miscellaneous*	Private Provider	9.5
	Perù	March 16, 2019 – April 30, 2019	March 16, 2020 – April 30, 2020	Miscellaneous*	Private Provider	9.5
	Perù	March 16, 2019 – June 30, 2019	March 16, 2020 – June 30, 2020	Miscellaneous*	Private Provider	9.5
	Perù	March 16, 2019 – June 30, 2019	March 16, 2020 – June 30, 2020	Miscellaneous*	Private Provider	9.5
	Brazil	March 16, 2019 – April 30, 2019	March 16, 2020 – April 30, 2020	Miscellaneous*	Public Provider	9.5
	Brazil	May 01, 2019 – May 31, 2019	May 01, 2020 – May 31, 2020	Miscellaneous*	Public Provider	9.5
	Brazil	June 01, 2019 – June 30, 2019	June 01, 20120 – June 30, 2020	Miscellaneous*	Public Provider	9.5
	Brazil	March 16, 2019 – April 30, 2019	March 16, 2020 – April 30, 2020	Miscellaneous*	Public Provider	9.5
	Brazil	May 01, 2019 – May 31, 2019	May 01, 2020 – May 31, 2020	Miscellaneous*	Public Provider	9.5
	Brazil	June 01, 2019 – June 30, 2019	June 01, 20120 – June 30, 2020	Miscellaneous*	Public Provider	9.5
	Brazil	March 16, 2019 – April 30, 2019	March 16, 2020 – April 30, 2020	Miscellaneous*	Public Provider	9.5
	Brazil	May 01, 2019 – May 31, 2019	May 01, 2020 – May 31, 2020	Miscellaneous*	Public Provider	9.5
	Brazil	June 01, 2019 – June 30, 2019	June 01, 20120 – June 30, 2020	Miscellaneous*	Public Provider	9.5
	Brazil	March 16, 2019 – June 30, 2019	March 16, 2020 – June 30, 2020	Miscellaneous*	Public Provider	9.5
(46)	Brazil	March 1, 2019 – May 31, 2019	March 1, 2020 – May 31, 2020	Miscellaneous*	Brazil's National Health System	9
	Brazil	March 1, 2019 – May 31, 2019	March 1, 2020 – May 31, 2020	Miscellaneous*	Brazil's National Health System	9
(47)	France	April 1, 2019 – May 31, 2019	April 1, 2020 – May 31, 2020	Miscellaneous*	University Hospital and Godinot Cancer Institute in Reims, Cancer Coordination Center (3C), Pasteur Hospital, Colmar	9.5
	France	April 1, 2019 – May 31, 2019	April 1, 2020 – May 31, 2020	Miscellaneous*	University Hospital and Godinot Cancer Institute in Reims, Cancer Coordination Center (3C), Pasteur Hospital, Colmar	9.5

	France	April 1, 2019 – May 31, 2019	April 1, 2020 – May 31, 2020	Miscellaneous*	University Hospital and Godinot Cancer Institute in Reims, Cancer Coordination Center (3C), Pasteur Hospital, Colmar	9.5

*Observations with impute number of daily events in order to calculate the weighted variation.

Supplementary Figure 1 Representation of geographic areas of the studies included in the analysis for cancer treatment variation

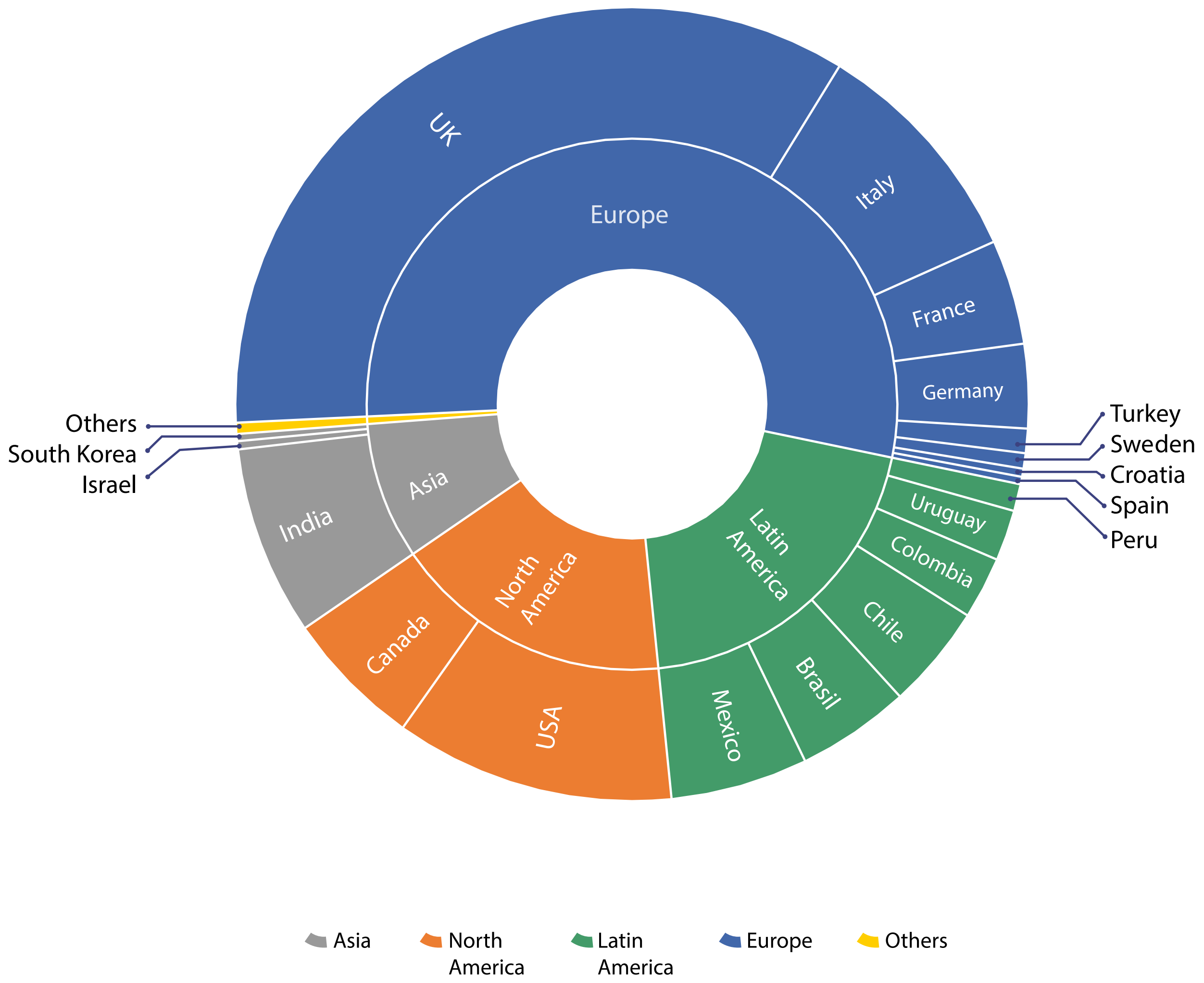

References

1. Ak N, Vatansever S. “Door to Treatment” Outcomes of Cancer Patients during the COVID-19 Pandemic. Chemotherapy. 2020;65(5–6):141–6.

2. Karacin C, Acar R, Bal O, Eren T, Sendur MAN, Acikgoz Y, et al. “Swords and Shields” against COVID-19 for patients with cancer at “clean” and “pandemic” hospitals: are we ready for the second wave? Support Care Cancer Off J Multinatl Assoc Support Care Cancer. 2021 Aug;29(8):4587–93.

3. Raj Kumar B, Pandey D, Rohila J, deSouza A, Saklani A. An observational study of the demographic and treatment changes in a tertiary colorectal cancer center during the COVID-19 pandemic. J Surg Oncol. 2020 Dec;122(7):1271–5.

4. Gazivoda V, Greenbaum A, Roshal J, Lee J, Reddy L, Rehman S, et al. Assessing the immediate impact of COVID-19 on surgical oncology practice: Experience from an NCI-designated Comprehensive Cancer Center in the Northeastern United States. J Surg Oncol. 2021 Jul;124(1):7–15.

5. Roberge D, Delouya G, Bohigas A, Michalowski S. Catching the Wave: Quantifying the Impact of COVID on Radiotherapy Delivery. Curr Oncol Tor Ont. 2020 Dec 25;28(1):152–8.

6. Challine A, Lazzati A, Katsahian S, Parc Y, Lefevre JH. Colorectal screening: We have not caught up. A surge of colorectal cancer after the coronavirus disease 2019 (COVID-19) pandemic? Surgery. 2021 Jul;170(1):349–50.

7. Allaix ME, Lo Secco G, Velluti F, De Paolis P, Arolfo S, Morino M. Colorectal surgery during the COVID-19 outbreak: do we need to change? Updat Surg. 2021 Feb;73(1):173–7.

8. Akhtar N, Rajan S, Chakrabarti D, Kumar V, Gupta S, Misra S, et al. Continuing cancer surgery through the first six months of the COVID-19 pandemic at an academic university hospital in India: A lower-middle-income country experience. J Surg Oncol. 2021 Apr;123(5):1177–87.

9. Cadili L, DeGirolamo K, McKevitt E, Brown CJ, Prabhakar C, Pao JS, et al. COVID-19 and breast cancer at a Regional Breast Centre: our flexible approach during the pandemic. Breast Cancer Res Treat. 2021 Apr;186(2):519–25.

10. Ranganathan P, Sengar M, Chinnaswamy G, Agrawal G, Arumugham R, Bhatt R, et al. Impact of COVID-19 on cancer care in India: a cohort study. Lancet Oncol. 2021 Jul;22(7):970–6.

11. Patt D, Gordan L, Diaz M, Okon T, Grady L, Harmison M, et al. Impact of COVID-19 on Cancer Care: How the Pandemic Is Delaying Cancer Diagnosis and Treatment for American Seniors. JCO Clin Cancer Inform. 2020 Nov;4:1059–71.

12. Martelli AJ, Machado RA, Pereira WM, Silveira DMM, da Cruz Perez DE, Martelli Júnior H. Impact of the COVID-19 pandemic in the head and neck cancer treatment in the Brazil. Oral Oncol. 2021 May;116:105148.

13. Castillo C, Camejo N, Amarillo D, Rodriguez F, Vitureira F, Krygier G, et al. Impact of the COVID-19 pandemic on health care activities at a Uruguayan mastology unit. J Cancer Res Ther. 2021 Jun;17(2):547–50.

14. Medas F, Ansaldo GL, Avenia N, Basili G, Bononi M, Bove A, et al. Impact of the COVID-19 pandemic on surgery for thyroid cancer in Italy: nationwide retrospective study. Br J Surg. 2021 Apr 30;108(4):e166–7.

15. Morris EJA, Goldacre R, Spata E, Mafham M, Finan PJ, Shelton J, et al. Impact of the COVID-19 pandemic on the detection and management of colorectal cancer in England: a population-based study. Lancet Gastroenterol Hepatol. 2021 Mar;6(3):199–208.

16. Furnari M, Eusebi LH, Savarino E, Petruzzellis C, Esposito G, Maida M, et al. Effects of SARS-CoV-2 emergency measures on high-risk lesions detection: a multicentre cross-sectional study. Gut. 2021 Jul;70(7):1241–3.

17. Diers J, Acar L, Baum P, Flemming S, Kastner C, Germer CT, et al. Fewer Operations for Cancer in Germany During the First Wave of COVID-19 in 2020–A Cohort Study and Time-Series Analysis. Dtsch Arzteblatt Int. 2021 Jul 12;118(27–28):481–2.

18. Lázaro Del Campo P, Ramírez López A, de la Cruz Benito B, de Paz Arias R, de Soto Álvarez T, Sánchez Vadillo I, et al. Hematopoietic cell transplantation during COVID-19 pandemic: experience from a tertiary hospital in Madrid. Expert Rev Hematol. 2021 Jan;14(1):1–5.

19. Pikkel YY, Duek OS, Ben Naftali Y, Link Y, Khayr M, Ullmann Y. Hidden in plain sight: the (other) danger of COVID-19. Melanoma Res. 2021 Aug 1;31(4):389–92.

20. Leite FPM, Curi C, Sanches SM, Curado MP, Fernandes GA, Moraes S, et al. How to maintain elective treatment of breast cancer during the COVID-19 pandemic-A cancer center experience. J Surg Oncol. 2021 Jan;123(1):9–11.

21. Vissio E, Falco EC, Collemi G, Borella F, Papotti M, Scarmozzino A, et al. Impact of COVID-19 lockdown measures on oncological surgical activity: Analysis of the surgical pathology caseload of a tertiary referral hospital in Northwestern Italy. J Surg Oncol. 2021 Jan;123(1):24–31.

22. Bravo JC, Navarro R, Rojas P, Hinrichs L, Schalper M, Zuñiga A, et al. Is Uro-oncological Surgery Safe During the COVID-19 Pandemic? Comparative Morbidity and Mortality in Patients Undergoing Surgery 2019-2020. Urol J. 2021 May 1;18(3):355–7.

23. Merchant J, Lindsey I, James D, Symons N, Boyce S, Jones O, et al. Maintaining Standards in Colorectal Cancer Surgery During the Global Pandemic: A Cohort Study. World J Surg. 2021 Mar;45(3):655–61.

24. Maspero M, Mazzola M, Bertoglio CL, Crippa J, Morini L, Magistro C, et al. Major cancer surgery during the coronavirus pandemic: experience from a tertiary referral center and COVID-19 hub in Northern Italy. Br J Surg. 2020 Sep;107(10):e440–1.

25. Penel N, Hammoudi A, Marliot G, De Courreges A, Cucchi M, Mirabel X, et al. Major impact of COVID-19 national containment on activities in the French northern comprehensive cancer center. Med Oncol Northwood Lond Engl. 2021 Feb 17;38(3):28.

26. S S, Sa H, M SK. Managing cancer during COVID pandemic - Experience of a tertiary cancer care center. Eur J Surg Oncol J Eur Soc Surg Oncol Br Assoc Surg Oncol [Internet]. 2021 May [cited 2022 Jul 21];47(5). Available from: https://pubmed.ncbi.nlm.nih.gov/33039295/

27. Franceschini G, Sanchez AM, Scardina L, Terribile D, Franco A, D’Archi S, et al. Mastectomy with immediate breast reconstruction during “phase 1” COVID-19 emergency: An Italian experience. Breast J. 2021 Jan;27(1):80–1.

28. Palluzzi E, Corrado G, Marchetti C, Bolomini G, Vertechy L, Bottoni C, et al. Medical treatment of patients with gynecologic cancer during the COVID-19 pandemic. Int J Gynecol Cancer Off J Int Gynecol Cancer Soc. 2021 Aug;31(8):1154–8.

29. Bertolaccini L, Sedda G, Spaggiari L. Paying Another Tribute to the COVID-19 Pandemic: The Decrease of Early Lung Cancers. Ann Thorac Surg. 2021 Mar;111(3):745–6.

30. Rich H, Jones B, Malin I, Hemington-Gorse SJ, Cubitt JJ. Plastic surgical management of skin cancer patients during the COVID-19 pandemic. J Plast Reconstr Aesthetic Surg JPRAS. 2021 Mar;74(3):644–710.

31. Fallara G, Sandin F, Styrke J, Carlsson S, Lissbrant IF, Ahlgren J, et al. Prostate cancer diagnosis, staging, and treatment in Sweden during the first phase of the COVID-19 pandemic. Scand J Urol. 2021 Jun;55(3):184–91.

32. Montesi G, Di Biase S, Chierchini S, Pavanato G, Virdis GE, Contato E, et al. Radiotherapy during COVID-19 pandemic. How to create a No fly zone: a Northern Italy experience. Radiol Med (Torino). 2020 Jun;125(6):600–3.

33. Gonnelli A, Montrone S, Cocuzza P, Ursino S, Manfredi B, Mattioni R, et al. Radiotherapy in the COVID-19 Pandemic Era. Vivo Athens Greece. 2020 Dec;34(6):3731–4.

34. Schiavone D, Torresan F, Rossi GP, Iacobone M. Reduced adrenal surgery in COVID-19 pandemic: a possible ticking time bomb. Br J Surg. 2021 Jul 23;108(7):e239–40.

35. Roseleur J, Gonzalez-Chica DA, Emery J, Stocks NP. Skin checks and skin cancer diagnosis in Australian general practice before and during the COVID-19 pandemic, 2011-2020. Br J Dermatol. 2021 Oct;185(4):853–5.

36. Gathani T, Clayton G, MacInnes E, Horgan K. The COVID-19 pandemic and impact on breast cancer diagnoses: what happened in England in the first half of 2020. Br J Cancer. 2021 Feb;124(4):710–2.

37. Clark JJ, Dwyer D, Pinwill N, Clark P, Johnson P, Hackshaw A. The effect of clinical decision making for initiation of systemic anticancer treatments in response to the COVID-19 pandemic in England: a retrospective analysis. Lancet Oncol. 2021 Jan;22(1):66–73.

38. Spencer K, Jones CM, Girdler R, Roe C, Sharpe M, Lawton S, et al. The impact of the COVID-19 pandemic on radiotherapy services in England, UK: a population-based study. Lancet Oncol. 2021 Mar;22(3):309–20.

39. Kvolik Pavić A, Zubčić V, Kvolik S. Workload changes during the COVID-19 pandemic and effects on the flow of cancer patients in the Maxillofacial Surgery Department. Med Glas Off Publ Med Assoc Zenica-Doboj Cant Bosnia Herzeg. 2021 Feb 1;18(1):133–7.

40. Perrone AM, Dondi G, Giunchi S, De Crescenzo E, Boussedra S, Tesei M, et al. COVID-19 free oncologic surgical hub: The experience of reallocation of a gynecologic oncology unit during pandemic outbreak. Gynecol Oncol. 2021 Apr;161(1):89–96.

41. Danesh MJ, Porter M, Brag K, Salian P, Olbricht S. COVID-19 impacts on dermatologic surgery patients: A single institution experience. J Am Acad Dermatol. 2021 Jun;84(6):1698–9.

42. Lee S, Heo J. COVID-19 pandemic: a new cause of unplanned interruption of radiotherapy in breast cancer patients. Med Oncol Northwood Lond Engl. 2021 Nov 5;39(1):5.

43. Giuliani J, Bonetti A. COVID-19 and cancer: A clear change not only in daily clinical practice but also in clinical research management. J Med Virol. 2021 May;93(5):2564–5.

44. Meggetto O, Jembere N, Gao J, Walker MJ, Rey M, Rabeneck L, et al. The impact of the COVID-19 pandemic on the Ontario Cervical Screening Program, colposcopy and treatment services in Ontario, Canada: a population-based study. BJOG Int J Obstet Gynaecol. 2021 Aug;128(9):1503–10.

45. Vázquez Rosas T, Cazap E, Delgado L, Ismael J, Bejarano S, Castro C, et al. Social Distancing and Economic Crisis During COVID-19 Pandemic Reduced Cancer Control in Latin America and Will Result in Increased Late-Stage Diagnoses and Expense. JCO Glob Oncol. 2021 May;7:694–703.

46. Fonseca GA, Normando PG, Loureiro LVM, Rodrigues REF, Oliveira VA, Melo MDT, et al. Reduction in the Number of Procedures and Hospitalizations and Increase in Cancer Mortality During the COVID-19 Pandemic in Brazil. JCO Glob Oncol. 2021 Jan;7:4–9.

47. Brugel M, Carlier C, Essner C, Debreuve-Theresette A, Beck MF, Merrouche Y, et al. Dramatic Changes in Oncology Care Pathways During the COVID-19 Pandemic: The French ONCOCARE-COV Study. The Oncologist. 2021 Feb;26(2):e338–41.
